# Supplementary material for: Impact of FLT3-ITD Insertion Length on Outcomes in Acute Myeloid Leukemia: A Propensity Score-Adjusted Cohort Study
Source: Biology (Basel). 2022 Jun 15;11(6):916. doi: 10.3390/biology11060916 (PMC9219926; doi:10.3390/biology11060916)
Supplement: Supplementary file 1 [file biology-11-00916-s001.zip › biology-1677146-supplementary.pdf]

Supplemental Figures

Figure S1: Loveplot describing covariate balance before and after matching for different insertion lengths of *FLT3* mutated AML for OS

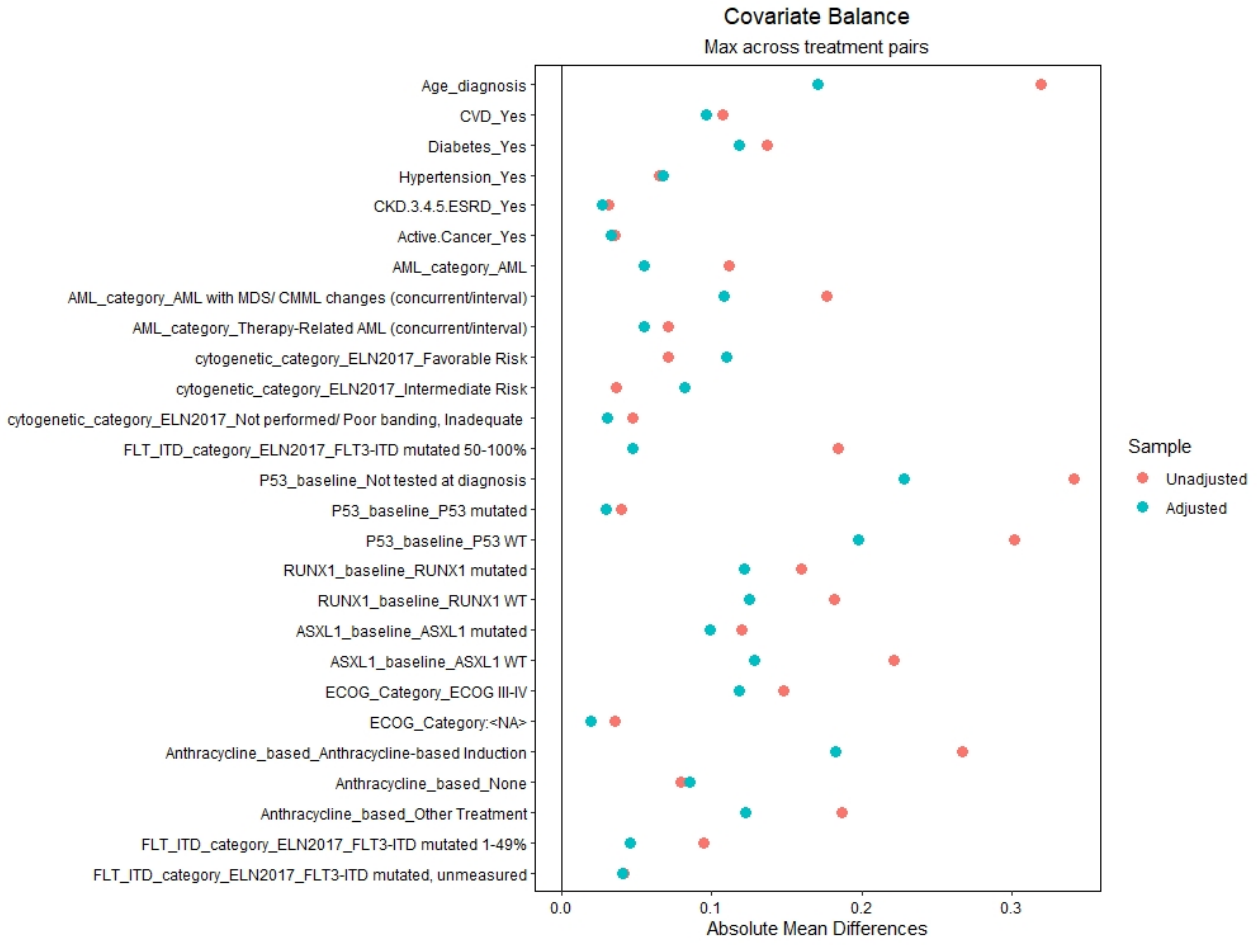

Figure S2. Loveplot describing covariate balance before and after matching for different insertion lengths of *FLT3* mutated AML for EFS

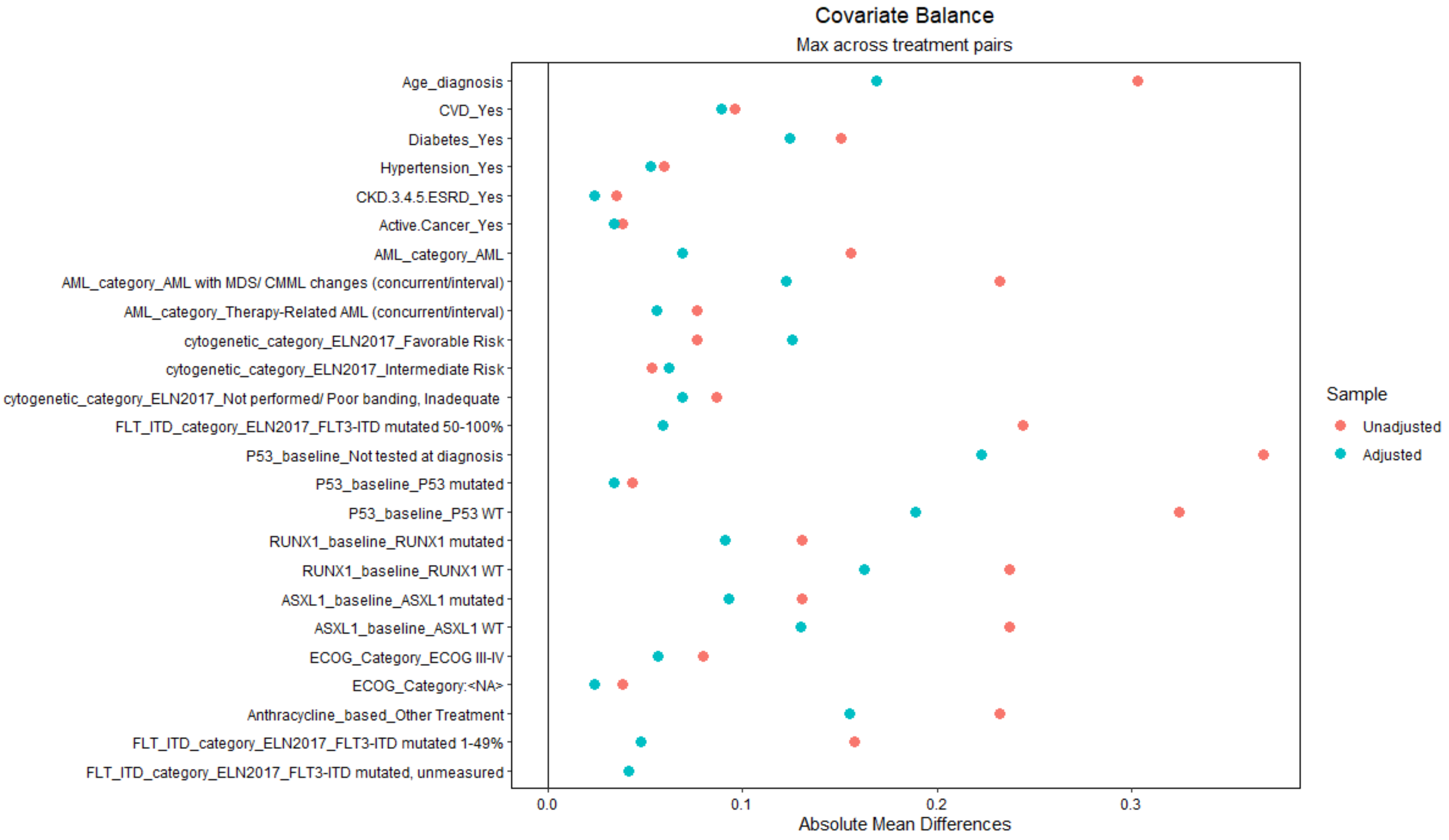

Figure S3: Loveplot describing covariate balance before and after matching for different insertion lengths of *FLT3* mutated and WT AML for OS

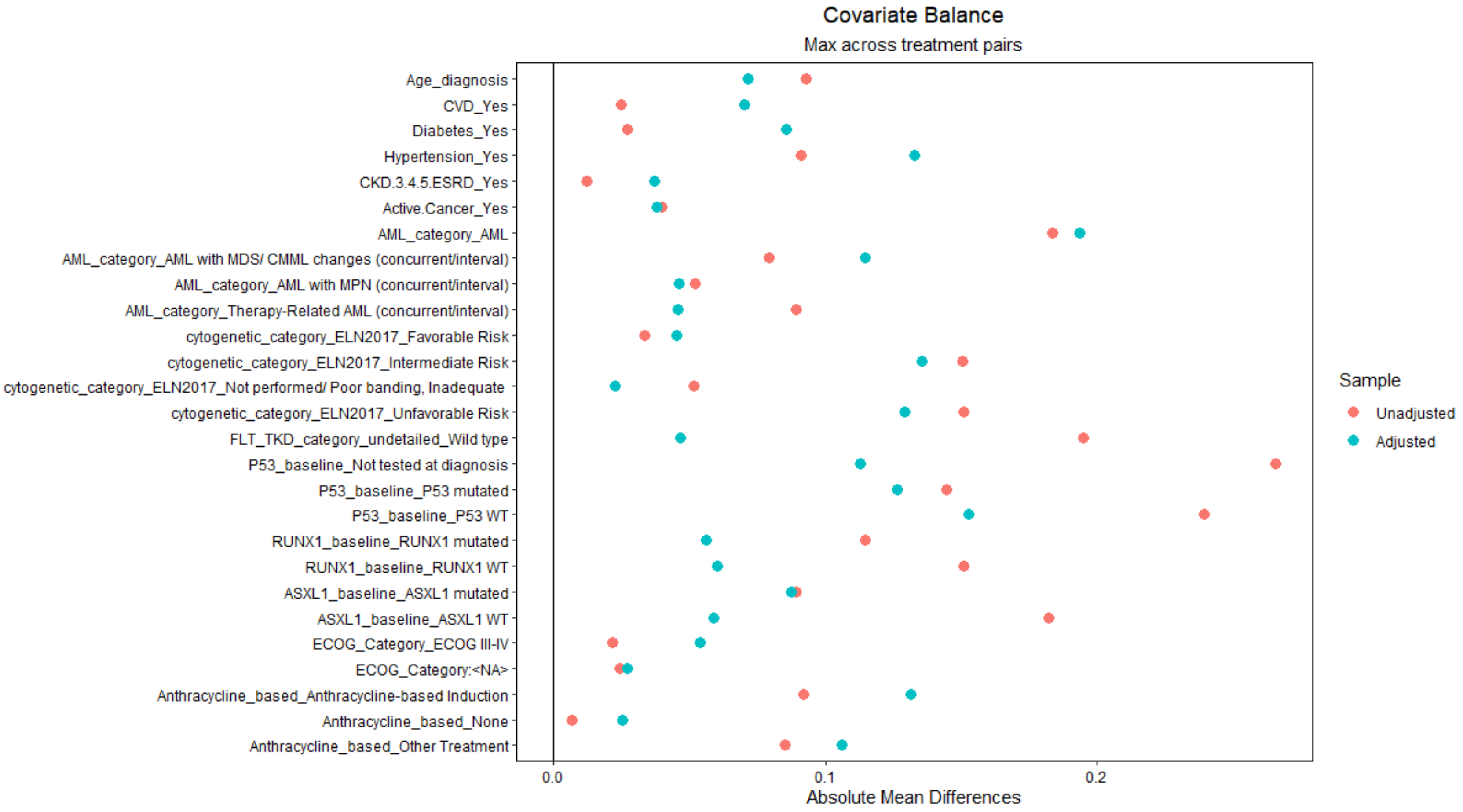

Figure S4: Propensity score-adjusted OS for patients with *FLT3*-ITD mutated and WT AML, categorized by insertion length

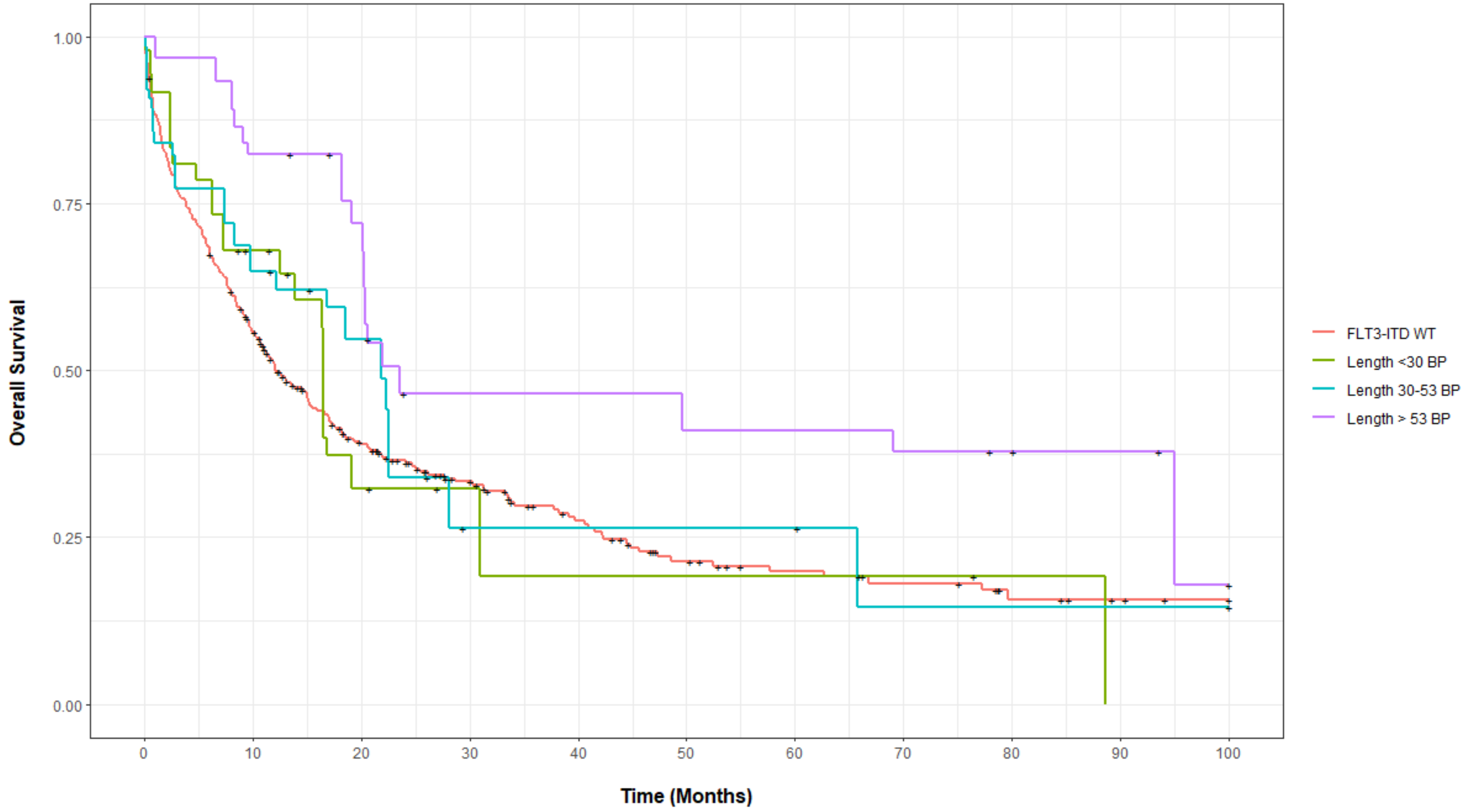

Figure S5: Unadjusted OS for patients with FLT3-ITD mutations, categorized by domain insertion expansion categories

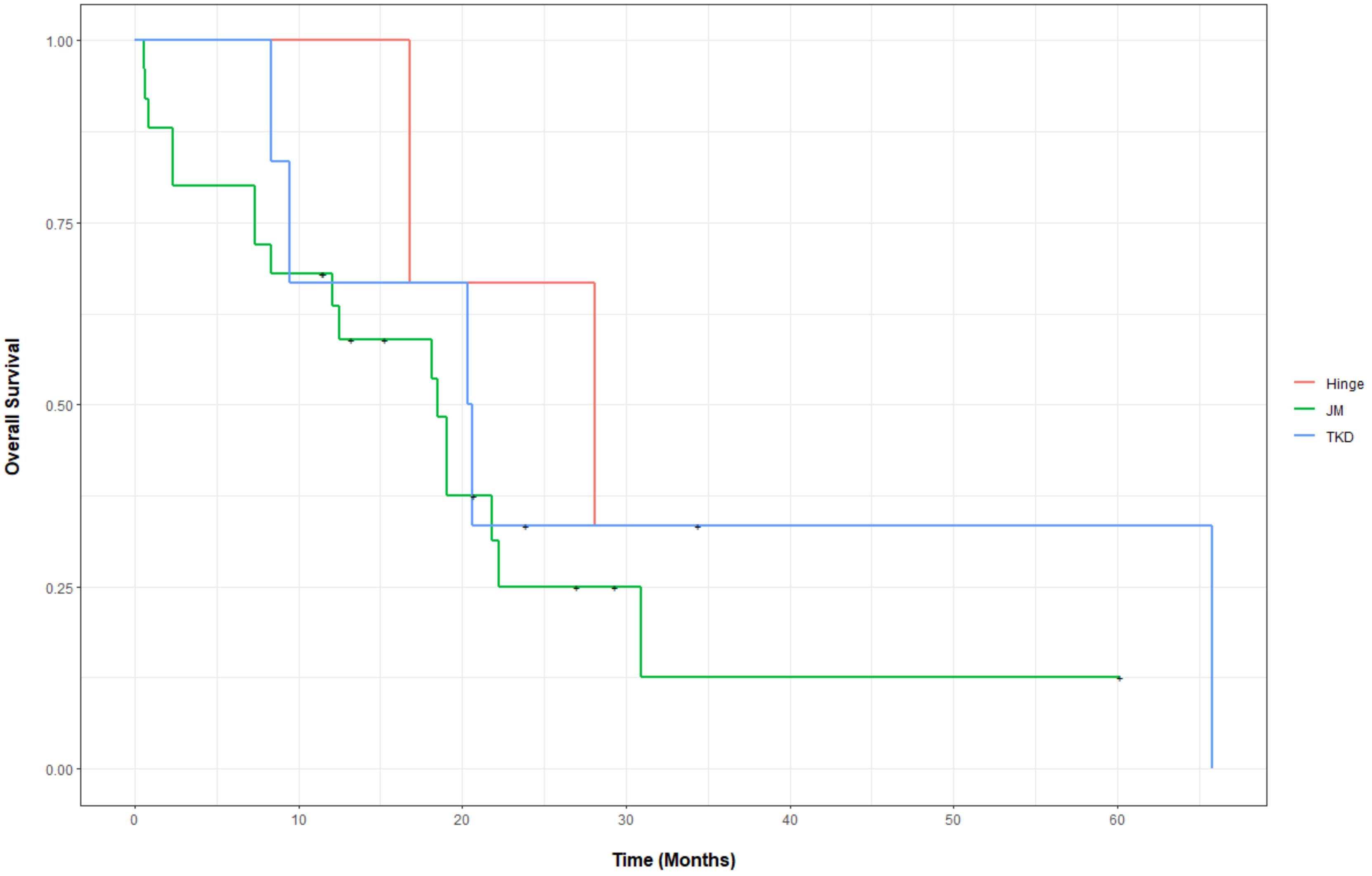

Figure S6. Unadjusted OS for patients with FLT3-ITD mutated AML treated with Midostaurin, categorized by insertion length

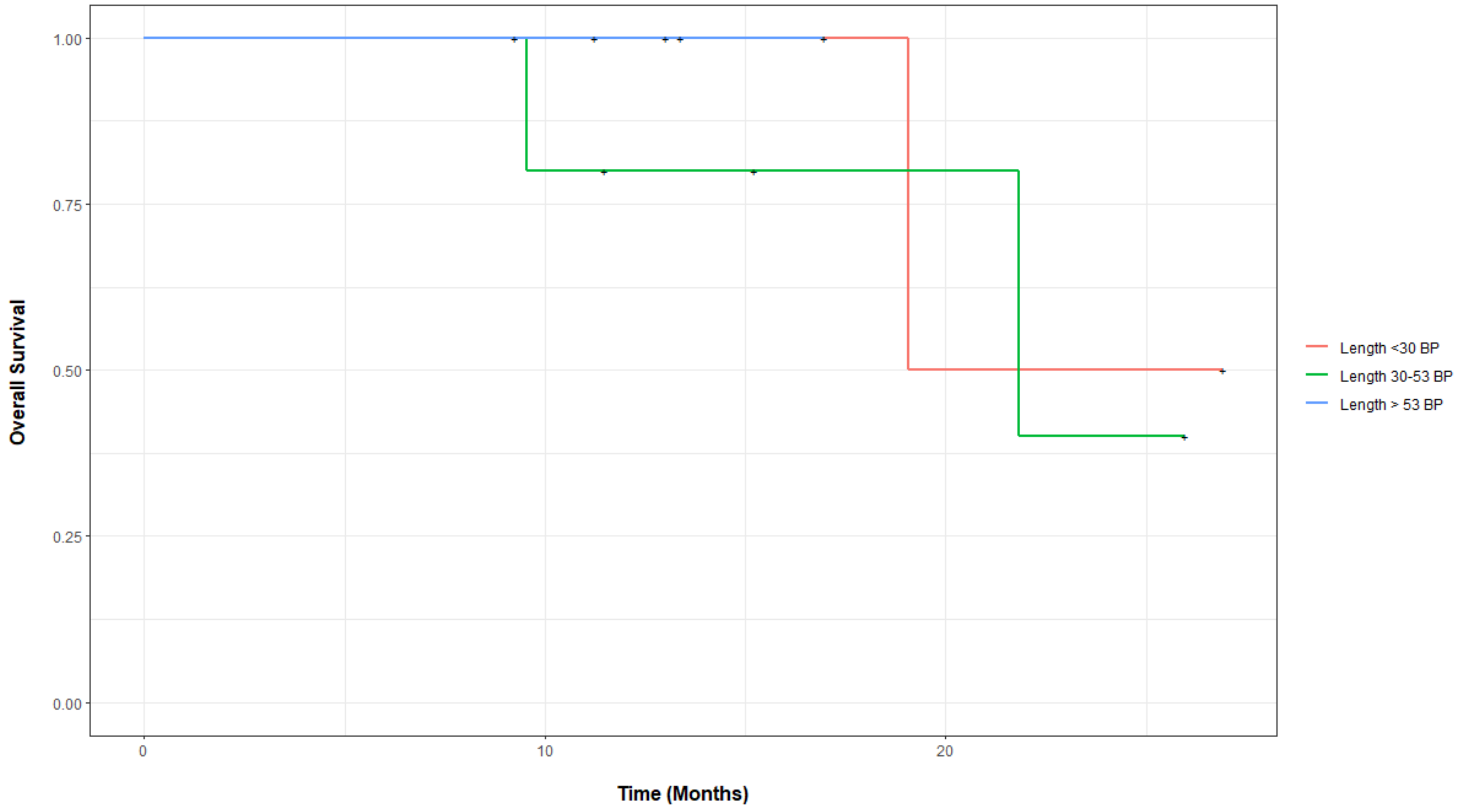

Figure S7: Unadjusted OS for patients with relapsed, refractory FLT3-ITD mutated AML treated with Gilteritinib, categorized by insertion length

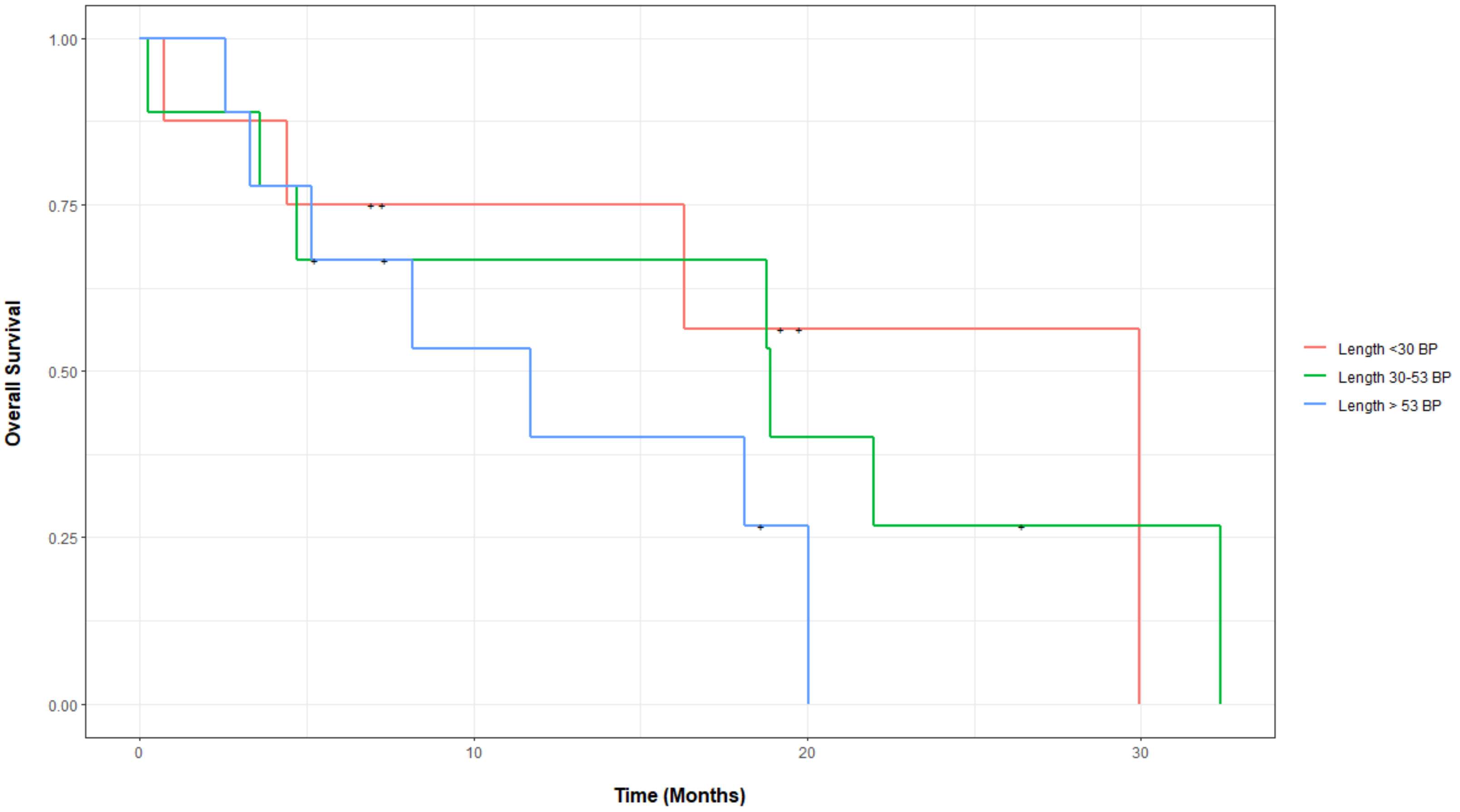
Overall Survival
1.00
0.75
0.50
0.25
0.00
0
10
20
30
Time (Months)
Length <30 BP
Length 30-53 BP
Length > 53 BP

Figure S8. Unadjusted OS for patients with *FLT3*-ITD and *NPM1* mutated and WT AML, categorized by insertion length

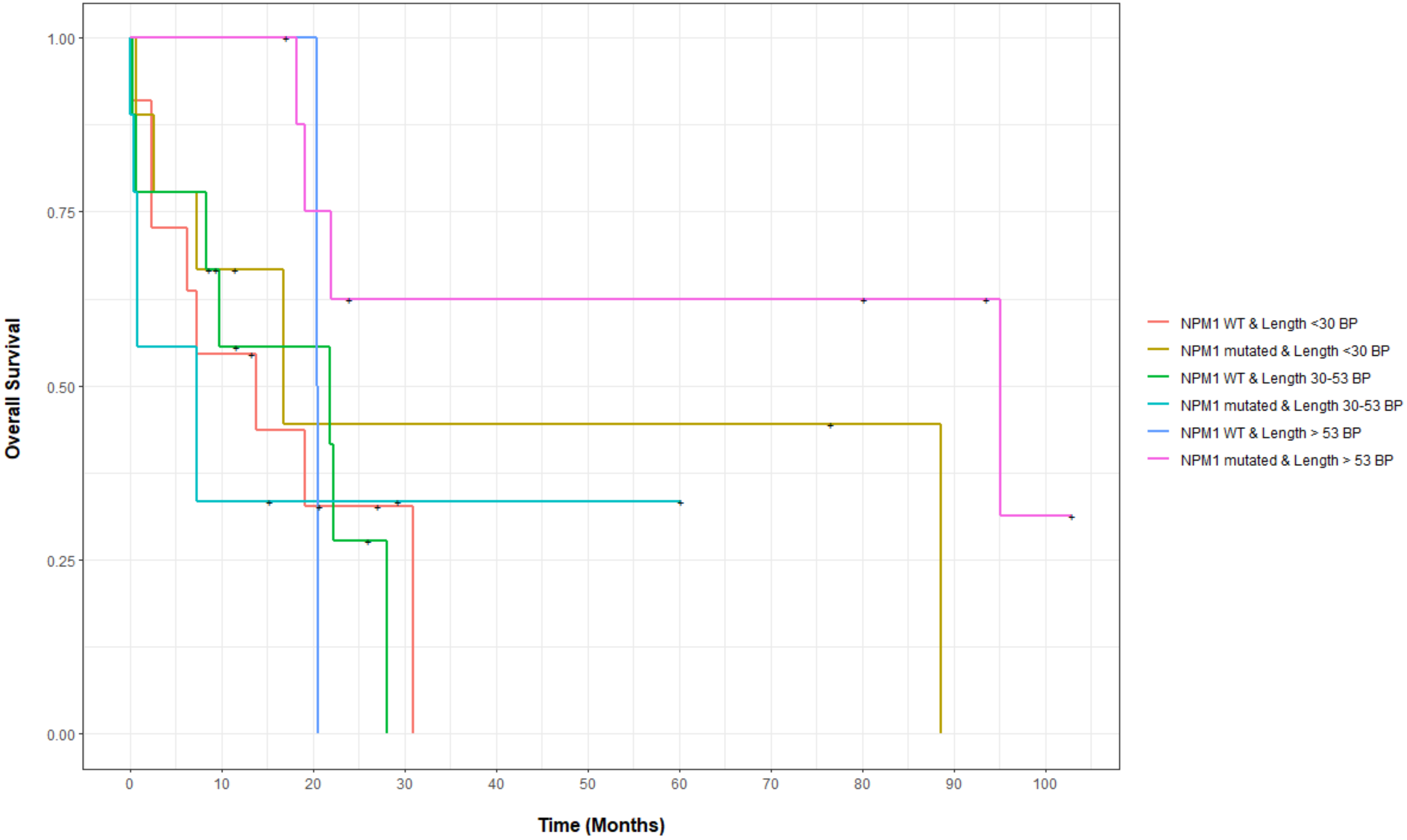

Supplemental Tables

Table S1. Adjusted OS at years 1-2 for patients with *FLT3*-ITD mutated AML, categorized by insertion length

	strata	time	Survival	Lower CI	Upper CI
1	Length <30 BP	12	0.66	0.50	0.88
2	Length <30 BP	24	0.27	0.13	0.60
3	Length 30-53 BP	12	0.60	0.44	0.82
4	Length 30-53 BP	24	0.33	0.17	0.64
5	Length >53 BP	12	0.75	0.58	0.95
6	Length >53 BP	24	0.41	0.24	0.69

Table S2. Adjusted EFS at years 1-2 for patients with *FLT3*-ITD mutated AML, categorized by insertion length

	strata	time	Survival	Lower CI	Upper CI
1	Length <30 BP	12	0.44	0.26	0.76
2	Length <30 BP	24	0.17	0.06	0.46
3	Length 30-53 BP	12	0.32	0.16	0.62
4	Length 30-53 BP	24	0.18	0.07	0.47
5	Length >53 BP	12	0.38	0.22	0.66
6	Length >53 BP	24	0.31	0.16	0.60

Table S3. Unadjusted median OS in months for patients with *FLT3*-ITD and *NPM1* mutated AML, categorized by insertion length

	strata	Median (months)	Lower CI	Upper CI	P-Value
Unadjusted					
1	*NPM1* WT & <30 BP	13.8	6.2	NA	0.2
2	*NPM1* mutated & <30 BP	16.8	7.3	NA	
3	*NPM1* WT & 30-53 BP	21.8	8.3	NA	
4	*NPM1* mutated & 30-53 BP	7.3	0.77	NA	
5	*NPM1* WT & >53 BP	20.5	20.4	NA	
6	*NPM1* mutated & >53 BP	95.1	21.9	NA	

Table S4. Unweighted multivariable cox proportional hazards for relative mortality of patients with *FLT3*-ITD and *NPM1* mutated AML

	HR	Lower CI	Upper CI	P-Value
ITD length[α]	0.99	0.96	1.0	0.34
Age at diagnosis	1.05	1.02	1.1	<0.01
TP53 WT	0.21	0.02	1.8	0.15
NPM1 WT	0.96	0.26	3.5	0.94
ITD length : *NPM1* WT interaction	1.0	0.98	1.04	0.41

[α] ITD Length: modeled as a continuous variable.

Table S5. Unweighted multivariable cox proportional hazards for relative mortality of patients with *FLT3*-ITD and *NPM1* mutated AML

	HR	Lower CI	Upper CI	P-Value
Length 30-53 BP [α]	1.6	0.49	5.5	0.42
Length >53 BP [α]	0.48	0.11	2.1	0.33
Age at diagnosis	1.1	1.02	1.1	<0.01
TP53 WT	0.24	0.03	2.1	0.20
NPM1 WT	1.70	0.53	5.5	0.36
Length 30-53 BP : *NPM1* WT	0.51	0.10	2.6	0.41
Length >53 BP : *NPM1* WT	1.4	0.16	12.1	0.77

[α] Compared to Length <30 BP.
